# Treatment of Chronic Ulcer of the Leg

**Published:** 1904-09

**Authors:** 


### Treatment of Chronic Ulcer of the Leg.

Horatio W. A. Cowan, (*Lancet*, London, Eng., July 2, 1904,) says that the beginning of the present year he was called to a woman aged 54 years, who had a chronic sloughing ulcer for twenty-two years situated on the outside of the left leg, some ten inches long and three inches wide, with indurated edges and some thrombosis of the veins of the inside of the knee. Having first cleansed the ulcer with charcoal poultices for two days, he applied wet butter cloth and then spread antiphlogistine over it after which cotton wool and a bandage were put on. This was done every day by the patient's friends for four months. The ulcer is now quite healed over and the induration is all gone. She is able to resume her ordinary housework. Cowan adds: "I publish this case in the hope that it might be useful to others as Unna's paste and all sorts of methods had been previously tried. I may say that I have no personal interest in antiphlogistine."

